# Effect of Housing Conditions on Cortisol and Body Fat Levels in Female Rhesus Macaques

**DOI:** 10.3390/biology10080744

**Published:** 2021-08-03

**Authors:** Dian G. M. Zijlmans, Lisette Meijer, Marit K. Vernes, Jacqueline A. M. Wubben, Linda Hofman, Annet L. Louwerse, Elisabeth H. M. Sterck, Jan A. M. Langermans, Marieke A. Stammes

**Affiliations:** 1 Biomedical Primate Research Centre, 2288 GJ Rijswijk, The Netherlands; meijer@bprc.nl (L.M.); vernes@bprc.nl (M.K.V.); wubben@bprc.nl (J.A.M.W.); linho1979@gmail.com (L.H.); louwerse@bprc.nl (A.L.L.); e.h.m.sterck@uu.nl (E.H.M.S.); langermans@bprc.nl (J.A.M.L.); stammes@bprc.nl (M.A.S.); 2 Animal Behaviour and Cognition, Department of Biology, Utrecht University, 3508 TB Utrecht, The Netherlands; 3 Department Population Health Sciences, Unit Animals in Science & Society, Faculty of Veterinary Medicine, Utrecht University, 3584 CM Utrecht, The Netherlands

**Keywords:** adiposity, animal welfare, cortisol, CT, housing, macaques

## Abstract

**Simple Summary:**

Macaques are highly social animals and commonly used in biomedical research. These studies often require group-living animals to be pair-housed in a controlled environment. This controlled environment likely affects stress and body fat levels. This study investigates the effect of a change from group- to pair-housing on cortisol, as measure for stress, and body fat levels of 32 female rhesus macaques. Besides individual differences, cortisol levels were higher in pair-housing compared to group-housing. Body fat levels did not differ between housing conditions. Accordingly, there was no clear association between cortisol and body fat levels.

**Abstract:**

Macaques are among the most commonly used non-human primates in biomedical research. They are highly social animals, yet biomedical studies often require group-living animals to be pair-housed in a controlled environment. A change in environment causes only short-term stress in adapting individuals, while non-adapting animals may experience long-term stress that can adversely affect study results. Individuals likely differ in their ability to adapt depending on individual characteristics. Changes in cortisol and body fat levels may reflect these different individual responses. Here, we investigate the long-term effect of a change from group- to pair-housing on cortisol and body fat levels in 32 female rhesus macaques, exploring whether age, dominance rank, original cortisol, and body fat levels are related to long-term stress in pair-housing. Hair samples were analyzed for cortisol levels, while anthropometric measurements and computed tomography were performed to quantify body fat. Monkeys served as their own control with a 7.5-month period between the measurements. Cortisol levels increased, while average body fat levels did not differ when individuals were moved from group- to pair-housing. Cortisol and body fat levels were not significantly correlated. Changes in cortisol were independent of age and dominance rank, whereas individual variation in body fat alterations was related to the group-housed body fat level and dominance rank. Although this study did not identify individual characteristics related to long-term stress in pair-housing, the individual variation confirms that some individuals are more resilient to change than others and provides possibilities for future refinement studies.

## 1. Introduction

Macaques are among the most commonly used non-human primates (NHPs) in biomedical research due to their high level of similarity to humans [1,2]. The regulations around the use of NHPs as animal models are strict. All studies need to comply with the principles of the 3Rs: replacement, reduction, and refinement [3], and welfare of the animals must be ensured as well as possible. Since the expression of natural behavior is a commonly used indicator of animal welfare, housing conditions should provide possibilities to perform species-specific natural behavior [4,5]. Macaques are highly social animals and have the behavioral need to engage in complex social interactions. Furthermore, the physical environment has to meet certain requirements concerning cage size, cage furniture, and enrichment [5,6,7,8]. Large enclosures with natural substrate and environmental enrichment lead to more natural and less stereotypic behavior [9,10,11,12]. Optimal housing conditions for macaques thus consist of naturalistic group-housing in large enclosures that mimic their natural habitat. However, these conditions are generally not feasible in biomedical research, since this requires a more controlled environment [13,14,15]. As a result, NHPs in biomedical studies are usually pair-housed in smaller cages in an indoor facility [13,16].

When animals are selected for experiments, they usually move from their larger groups to pair-housing in this controlled environment. The effect of relocations on primate physiology and behavior have been extensively studied. Relocations have been reported to cause stress, i.e., they lead to alterations in body weight, a suppressed immune response, increased cortisol levels, increased heartrate, and behavioral changes [14,17,18,19,20,21]. These changes are often temporary, consistent with short-term stress, which implies that the individuals are able to adapt to their new situation [22]. Unfortunately, some animals remain stressed for an extended period and experience long-term stress, as their physiological parameters and behavior do not return to baseline levels [14,23,24]. This long-term stress indicates that these animals have difficulty to adapt to their new environment and, subsequently, this may adversely affect study results. These differences between individuals in their ability to adapt suggest that some individuals are more resilient than others, and this may depend on individual characteristics, such as age and dominance rank [22,25,26,27].

Measuring the biomarkers mentioned above for stress can be divided as invasive and non-invasive. Invasive measurements, which require blood sampling or telemetry, will likely have its impact on stress too. Non-invasive biomarkers include hair cortisol concentrations (HCCs) and body weight, which is monitored anyway to follow course of growth, development, and basic health. Long-term stress can be reliably measured in HCCs, as stressful events can be found back in hair samples for at least 14 weeks [28]. During stress, the hypothalamic-pituitary-adrenal axis produces glucocorticoids such as cortisol. These have been associated with a suppression of the growth hormone system to metabolize fat [29,30]. Stress-induced changes in fat metabolism may increase body fat, especially in the abdominal region [31,32]. Evidence for increased body fat due to stress was found in several studies: social stress from subordination resulted in a high abdominal fat deposition in female long-tailed macaques (*Macaca fascicularis*) [30]. Similarly, the incidence of obesity increased when group composition was altered, inducing social stress, in male vervet monkeys (*Cercopithecus aethiops*) [29,33]. Thus, changes in HCCs and body fat levels may both serve as biomarkers for long-term stress and signal individual differences in adaptive ability.

The current study aims to identify individual characteristics associated with long-term stress resulting from change in housing conditions when individuals enter an experiment, thereby providing possibilities to refine selection procedures. This contributes to optimizing animal welfare of NHPs in biomedical studies, thereby increasing scientific validity of experimental results [13]. We therefore investigate the long-term effect of a change from group- to pair-housing on HCCs and body fat levels of captive female rhesus macaques (*Macaca mulatta*). As this study is based on an opportunistic data collection from another study, solely females are incorporated, and we only investigated the change in housing conditions from group-to pair-housing. Individuals served as their own control as they moved from group-housing encompassing indoor and outdoor enclosures to pair-housing in smaller indoor enclosures with a 7.5-month adaptation period. Hair samples were analyzed for HCCs, while anthropometric measurements and computed tomography (CT) were performed to quantify body fat. HCCs and body fat levels are expected to be higher in pair-housing compared to group-housing; and to be correlated.

## 2. Materials and Methods

### 2.1. Animals and Ethics

For this study, 32 female rhesus macaques housed at the Biomedical Primate Research Centre (BPRC) in Rijswijk, The Netherlands, were examined. BPRC is accredited by the Association for Assessment and Accreditation of Laboratory Animal Care (AAALAC International). All procedures performed in this study, as well as housing and husbandry, were in accordance with the European Directive 2010/63 and Dutch law. Ethical approval for this separate study was not required as the data were obtained as part of a tuberculosis study (animal license AVD5020020172645). As a result, animals were not randomly selected, as they had to fulfill several study criteria, including age, sex, and body composition. Animals were aged between 4 and 9 years old (mean = 6.3, SE = 0.2) and weighed between 5.4 and 10.1 kg (mean = 7.7, SE = 0.2) at the start of the study. An exclusion criterion was applied on maximum body weight for the animals to fit in the PET–CT. The tuberculosis study consisted of an adaptation and vaccination phase of 7.5 months after which the infectious challenge took place. Our study was performed during this initial 7.5-month period.

### 2.2. Housing and Husbandry

The initial housing conditions for 27 females consisted of group-housing, while five females had already been pair-housed for a longer period (at least nine months). This last group of animals was also relocated from one building to another but remained pair-housed in a similar enclosure. They served as a control group to test whether the changes in HCCs and body fat levels were related to the housing condition and not due to the relocation itself. In both housing conditions, the same light/dark cycles were applied with a 12/12 light/dark cycle from 7 to 19 h. Both conditions also had access to natural light, by the outside enclosure, or by the windows in the stable.

#### 2.2.1. Group-Housing

Group-housed individuals came from nine different groups, which were formed by adhering to natural group dynamics. Each group consisted of 15–40 individuals from several multigenerational matrilines and had free 24 h access to enriched indoor (72 m^2^ and 2.85 m high) and outdoor (250 m^2^ and 3.1 m high) enclosures. Both indoor and outdoor enclosures contained several compartments and visual barriers. The inside enclosure contained sawdust bedding, while the outside enclosure had a sand bedding with natural plant growth. The enclosures were equipped with several climbing structures, beams, perches, fire hoses, car tires, slides, and a swimming pool to stimulate natural behavior [34]. Drinking water was available *ad libitum* via automatic water dispensers. The monkeys were fed monkey chow (Ssniff, Soest, Germany) daily in the morning, complemented with fresh fruit, vegetables, or a grain mixture in the afternoon.

#### 2.2.2. Pair-Housing

After moving to the experimental facility, all individuals were pair-housed in four inside rooms with six enclosures per room. Most individuals of a pair were maternally related to each other (sisters, cousins, or nieces). One pair was not related but came from the same breeding group. Two pairs were formed by introducing unfamiliar females to each other: one formerly group-housed female was coupled to a formerly pair-housed female and two other formerly pair-housed females also formed a new pair. Ten pairs had access to a single enclosure, while six pairs had access to a double enclosure. A single enclosure is 2 m high with a surface of 2 m^2^. The enclosures were split in three height levels and contained visual barriers, perches, and fire hoses. Water was available *ad libitum* and monkeys were fed monkey chow daily around noon. Fresh fruit was provided in the morning and vegetables in the afternoon. Food enrichment was offered daily and additional non-food enrichment (toys) varied weekly according to a rotation schedule.

#### 2.2.3. Relocation

All females were relocated from their initial housing condition to pair-housing on two consecutive days. On the day of the relocation, animals were anesthetized with an intramuscular injection of ketamine (ketamine hydrochloride, Ketamine 10%; Alfasan, Woerden, The Netherlands, 100 mg/mL, 10 mg/kg) in combination with the α2-adrenoceptor agonist medetomidine (medetomidine hydrochloride, Sedastart; AST Farma, Oudewater, The Netherlands, 1 mg/mL, 0.05 mg/kg), which was reversed afterwards with atipamezole (atipamezole hydrochloride, Sedastop; AST Farma, Oudewater, The Netherlands, 5 mg/mL, 0.25 mg/kg). Hair was shaved from the posterior vertex region of the neck to analyze hair cortisol concentrations (HCCs). Anthropometric measurements and computed tomographies (CTs) of the abdominal area were performed to quantify body fat. These three procedures were repeated in exactly the same manner after 7.5 months in pair-housing.

### 2.3. Procedures

#### 2.3.1. Hair Cortisol Analysis

Hair was shaved from the posterior vertex region of the neck, packed in aluminum foil, and stored in a freezer at −20 °C. Hair samples were collected in the three months preceding relocation and at the day of the relocation, representing HCCs in group-housing. Hair samples were collected again during a required management procedure after roughly six months in pair-housing and after 7.5 months in pair-housing. This resulted in two hair samples per housing condition for each animal.

HCCs were measured from the samples as described by Davenport et al. [28]. In short, the hair was washed with isopropanol twice and allowed to dry for five days before being ground to powder in a beadbeater. Approximately 50 mg of the powder was incubated for 24 h in 1 mL methanol to extract the cortisol, followed by centrifugation. The supernatant was transferred into another tube and left to dry on a heating block for approximately 5.5 h. The dried cortisol extract was dissolved in 400 µL phosphate buffer and analyzed with an enzyme immunoassay kit (Salimetrics, State College, PA, USA) according to the manufacturer’s instructions. HCCs were corrected for powder weight by calculating the pg cortisol/mg hair. All samples were analyzed twice with an average coefficient of variation of 3.8%.

Since HCCs in the two samples of the same housing condition were highly correlated (group-housing: Spearman correlation, r = 0.825, n = 31, *p* < 0.0005; pair-housing: Spearman correlation, r = 0.809, n = 32, *p <* 0.0005), the average value for each housing condition was used in further analyses.

#### 2.3.2. Anthropometric Measurements

Anthropometric measurements were performed following a standard procedure [35]. Briefly, body weight was measured with a standard scale, crown-rump length (height) was measured with a measuring mat for human infants (Seca, Hamburg, Germany), and abdominal circumference was measured with a measuring tape. A Baseline Pro skinfold caliper was used to measure abdominal skinfold thicknesses at the height of the umbilicus. All measurements were performed three times and always by the same person (DGMZ). The average values of the three measurements were used in further analyses. A species-specific weight-for-height index for rhesus macaques, known as WHI3.0, was calculated as body weight (kg) divided by the third power of height (m) [35]. We will refer to this as WHI.

#### 2.3.3. CTs

To obtain CTs, the monkeys were positioned head first supine in a LFER 150 PET-CT (Mediso, Budapest, Hungary). A single cone beam scan of 360 projections was performed at the lumbar vertebra level at 80 kVp, 980 mA, and a scan time of 50 ms for each projection. For analysis, only the area between the third (L3) and the fifth (L5) lumbar vertebra was included. Three observers analyzed the scans together in VivoQuant 4.5rc4 (InviCRO, Boston, MA, USA). Fat was measured with a density range, because fat is a solid though flexible type of tissue, the shape and density of which are defined by the surrounding tissue.

Since abdominal fat deposition was found to have a significant impact on the well-being and more important the risk of developing diseases, we aimed to investigate both the subcutaneous fat and the abdominal fat found around the organs in the abdominal area [29]. This cannot be captured in a single value and therefore different Hounsfield unit (HU) ranges were used for abdominal (−170 HU to −90/−60/−30/0 HU) and subcutaneous fat tissue (−170 HU to 0/30/60/90/120 HU). One HU range for each fat tissue type did not suffice, as there was individual variation in the density of fat tissue between monkeys with more or less fat. The HU range included as much fat tissue as possible without covering any other tissue, such as muscles, intestines, or kidneys.

**Figure 1:**
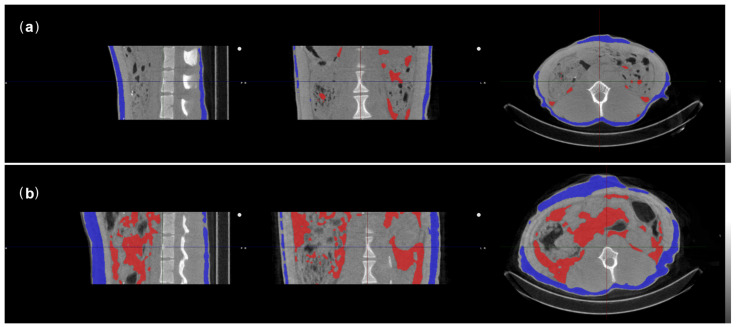
Representative CT sections of two female rhesus macaques with regions of interest defining the abdominal body fat (red) and subcutaneous body fat (blue). The three panels represent one animal visualized in sagittal, coronal, and transversal direction in which the center of the cross-hairs represents the same point in the three directions. The respective macaques had body weights of 6.45 kg (**a**) and 7.43 kg (**b**). (**a**) A lean individual with a WHI of 53.5 kg/m3 and a TBF of 10.1%, while (**b**) represents an individual with a relatively high amount of body fat (WHI = 62.6 kg/m3, TBF = 32.7%).

Next, abdominal fat tissue, subcutaneous fat tissue, and the body volume of the monkey were defined for each scan in mm^3^ (Figure 1). Abdominal and subcutaneous body fat percentages were calculated by dividing the amount of fat tissue by the body volume. When the monkeys had too little body fat to differentiate on CT, body fat percentage was set at 0% [36]. Total body fat percentage (TBF) was measured as the sum of abdominal and subcutaneous body fat percentages. All scans were randomized and re-analyzed by two of the observers separately three months later (LM, MAS). The level of agreement between the ratings was moderate (κ = 0.555) for abdominal body fat, while there was substantial agreement (κ = 0.666) for subcutaneous body fat [37]. The median value of the assessments was used for further analyses.

#### 2.3.4. Dominance Rank

Experienced ethologists and colony managers regularly recorded dominance rank in the colony and were able to categorize the females as being low-ranking (n = 16) or high-ranking (n = 11) in their social group before relocation. Dominance rank was evaluated again when the females were relocated and pair-housed as being low-ranking (n = 13) or high-ranking (n = 14) based on video analyses of agonistic behavior between the individuals of a pair. This approach resulted in 86% agreement with the subjective evaluation from the animal trainer (MKV).

### 2.4. Data Analyses

**Figure A1:**
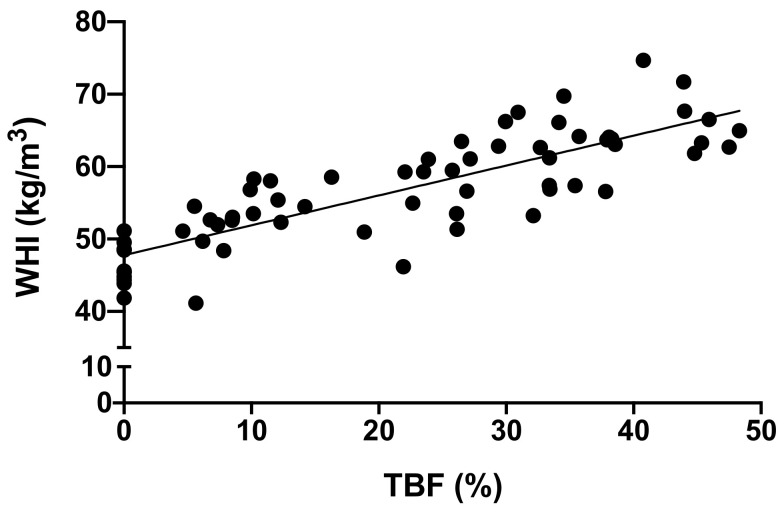
Weight−for−height index (WHI) plotted against total body fat percentage (TBF) in the abdominal region of female rhesus macaques in group− and pair−housing. n = 64.

Data analyses were performed in IBM SPSS Statistics v26. Anthropometric measures and body fat percentages from the CTs were highly correlated under both housing conditions (r > 0.784, n = 64, *p* < 0.0005; Table A1; Figure A1) and overall provided similar results (Table A2). We therefore only report statistical outcomes for WHI and TBF. HCCs and body fat levels were compared between group- and pair-housing with a paired samples t-test or Wilcoxon signed ranks test, depending on whether the data were normally distributed or not. Normal distribution of the data was visually checked using boxplots and histograms, as well as tested with the Shapiro-Wilk test. Pearson or Spearman correlations were used to test the relationship between HCCs and body fat levels. The delta values for HCC, WHI, and TBF were calculated by subtracting the values in pair-housing from group-housing (the baseline value).

Multiple linear regression analyses were used to check which individual factors, i.e., age and dominance rank, affected HCC and body fat levels of females that moved from group- to pair-housing in both housing conditions and also the changes in HCC and body fat. Normality and homoscedasticity of the residuals were visually checked for each model. These assumptions were violated in the models for pair-housed HCC and changes in HCC when including all individuals. The data contained one major outlier (pair-housed HCC = 150.8 pg/mg, delta HCC = 130.3 pg/mg), which was the only group-housed individual that was coupled to an unfamiliar female in pair-housing. We considered this a valid reason to exclude this individual from the cortisol analyses. After this individual was excluded, the assumptions of normality and homoscedasticity of the residuals were fulfilled. There was no collinearity between the variables in the models as the variance inflation factor was equal to or lower than 1.26 for all variables.

Initial housing condition could not be included in the models on the delta values due to collinearity. To check whether changes in HCC and body fat were related to the housing condition and not the relocation, an independent samples t-test or a Mann-Whitney U-test was performed on the delta values. Other potential confounding factors, i.e., number of sedations, number of reported injuries, exposure to humans, cage size (double or single enclosure), and relatedness between the individuals of a pair, were tested but did not significantly influence the outcome measures (Table A3). The outcomes were considered statistically significant at α = 0.05 and all tests were two-tailed.

## 3. Results

### 3.1. Group-Housing

**Figure 2:**
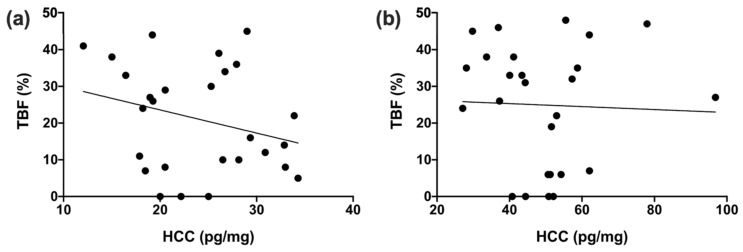
Total body fat percentage (TBF) in the abdominal area plotted against the hair cortisol concentration (HCC) of female rhesus macaques in group-housing (**a**) and pair-housing (**b**).

HCCs in group-housing were on average 24.0 pg/mg with little variation between individuals (SE: 1.2, range: 12.0–34.3 pg/mg). HCCs were independent of age (F (1,24) = 1.336, *p* = 0.259) and dominance rank (F (1,24) = 1.254, *p* = 0.274). TBF of group-housed individuals was on average 21.1% and there was considerable variation between individuals (SE: 2.8, range: 0–45.3%; Table A4). TBF and WHI were independent of dominance rank (F (1,24) = 0.041, *p* = 0.841; F (1,24) = 0.162, *p* = 0.691). Age had no significant effect on WHI (F (1,24) = 2.030, *p* = 0.167), but older individuals did have a higher TBF compared to younger macaques (F (1,24) = 6.145, *p* = 0.021). There was no significant correlation between HCC and WHI (Pearson correlation, r = −0.253, n = 27, *p* = 0.203) or between HCC and TBF (Pearson correlation, r = −0.272, n = 27, *p* = 0.171; Figure 2a) in group-housing.

### 3.2. Pair-Housing

Pair-housed individuals had average HCCs of 49.3 pg/mg and variation between individuals was higher compared to the variation in group-housing (SE: 3.0, range: 27.1–96.8 pg/mg). Dominance rank (F (1,23) = 0.961, *p* = 0.337) and age (F (1,23) = 0.000, *p* = 0.996) had no significant effect on HCCs in pair-housing. TBF was on average 24.8% and the variation between individuals was roughly similar compared to group-housing (SE: 3.1, range 0–48.3%; Table A4). TBF and WHI were independent of age (F (1,24) = 2.441, *p* = 0.131; F (1,24) = 1.493, *p* = 0.234) and dominance rank (F (1,24) = 0.000, *p* = 0.985; F (1,24) = 0.097, *p* = 0.758) in pair-housing. There was no significant correlation between HCC and WHI (Spearman correlation, r = −0.210, n = 26, *p* = 0.304) or between HCC and TBF (Spearman correlation, r = −0.053, n = 26, *p* = 0.798; Figure 2b) in pair-housing.

### 3.3. Comparison between Housing Conditions

**Figure 3:**
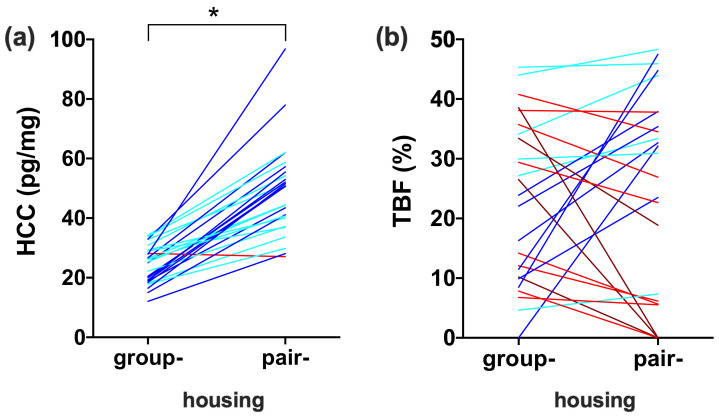
The effect of group- and pair-housing on hair cortisol concentrations (HCC; (**a**)) and total body fat percentage (TBF) in the abdominal region (**b**) of female rhesus macaques. Each line represents an individual. Dark blue lines indicate large relative increase, while light blue lines represent small relative increases. Similarly, dark red lines represent large decreases and normal red lines represent relatively small decreases. * *p* < 0.05.

HCCs increased for all individuals, except one when individuals were moved from group- to pair-housing (Figure 3a). HCCs significantly increased with an average of 112% (SE: 12, range: −4–247%; Wilcoxon signed ranks test, Z = −4.432, n = 26, *p* < 0.0005). Delta HCC was highly variable (SE: 2.7, range: −1.08–68.9 pg/mg) and independent of age (F (1,21) = 0.389, *p* = 0.540), group-housed HCC (F (1,21) = 0.378, *p* = 0.545), group-housed dominance rank (F (1,21) = 2.669, *p* = 0.117), and pair-housed dominance rank (F (1,21) = 1.826, *p* = 0.191). Delta HCC was not significantly related to delta WHI (Pearson correlation, r = −0.083, n = 26, *p* = 0.688) or delta TBF (Pearson correlation, r = −0.046, n = 26, *p* = 0.824). Delta HCC significantly differed between formerly group-housed (25.1 ± 2.7 pg/mg) and formerly pair-housed females (−5.7 ± 7.7 pg/mg; independent samples t-test, t = 4.400, n = 31, *p* < 0.0005), which confirms that the increase in HCC resulted from changing from group- to pair-housing and not from the relocation.

WHI and TBF did not significantly change when individuals moved from group- to pair-housing (paired samples t-test, t = 1.490, n = 27, *p* = 0.148; Wilcoxon signed ranks test, Z = −0.937, n = 27, *p* = 0.349). However, there was large individual variation in body fat alterations (Table A2; Figure 3b). Eight individuals lost more than 10% of their original body weight, while five individuals gained more than 10% of their original body weight. WHI increased in 13 individuals, while TBF increased in 15 individuals. For TBF, the average increase was 15.6% (SE: 3.2, range 0.6–39.0%) and the average decrease was 11.3% (SE: 3.1, range 0.3–38.5%).

**Figure 4:**
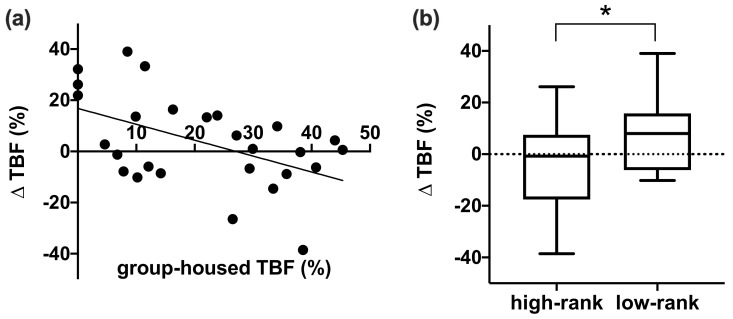
The effect of group-housed total body fat percentage (**a**) and group-housed dominance rank (**b**) on change in total body fat percentage (ΔTBF). * *p* < 0.05.

Delta WHI was independent of age (F (1,22) = 0.367, *p* = 0.551) and pair-housed dominance rank (F (1,22) = 0.440, *p* = 0.514). WHI decreased in individuals with a high group-housed WHI, while WHI generally increased in individuals with a low group-housed WHI (F (1,22) = 4.487, *p* = 0.046). There was also a trend for individuals with a high rank in their social group to decrease WHI in pair-housing, while low-ranking individuals increased their WHI after the relocation (F (1,22) = 3.409, *p* = 0.078). A similar pattern was found for TBF. Neither age (F (1,22) = 0.955, *p* = 0.339) nor pair-housed dominance rank (F (1,22) = 0.010, *p* = 0.923) influenced alterations in TBF. TBF increased in individuals with low group-housed TBF (F (1,22) = 9.373, *p* = 0.006; Figure 4a) and dominance rank (F (1,22) = 4.282, *p* = 0.050; Figure 4b), while TBF decreased in individuals with high group-housed TBF and dominance rank. Formerly group-housed and formerly pair-housed individuals did not significantly differ in delta WHI (independent samples t-test, t = −1.040, n = 32, *p* = 0.307) or delta TBF (Mann-Whitney U-test, U = 62, n = 32, *p* = 0.775).

## 4. Discussion

This study investigated the effect of moving female rhesus macaques from group- to pair-housing on hair cortisol concentrations (HCCs) and body fat levels and aimed to identify individual characteristics associated with long-term stress in pair-housing. When individuals moved from group- to pair-housing, HCCs increased for all individuals, except one, while average weight-for-height index (WHI) and total body fat percentage (TBF) did not change. Delta HCC was independent of age, dominance rank, and group-housed HCC, while alterations in body fat levels were related to the group-housed body fat level and dominance rank. However, HCCs and body fat levels were not significantly correlated.

This study used HCCs as a biomarker for long-term stress. Hair sampling is minimally invasive and provides a reliable method for the measurement of long-term cortisol [28,38]. Nevertheless, several points need to be considered when using hair samples, e.g., location on the body, amount of sunlight, repeated sampling, and season [38,39]. To minimize the effect of these possible confounders, we standardized the location of the hair sample at the posterior vertex region of the neck and collected all samples at the same time points.

In the current study, HCCs increased from 24.0 pg/mg in group-housing to 49.3 pg/mg in pair-housing. These values should be placed in perspective to other publications. Semi-free ranging adult female rhesus macaques have mean HCCs of 44.0 pg/mg, while individually housed male rhesus macaques have mean HCCs of 110.3 pg/mg [28,40]. HCCs of group-housed male and female rhesus macaques are population density-dependent: young adults (5–9 year) in a low-density population have mean HCCs of 37.7 pg/mg, while HCCs are on average 77.7 pg/mg in the high-density populations [41]. Housing conditions thus have a strong effect on HCCs and HCCs are generally higher in smaller and more crowded environments. Early life social experience in naturalistic groups may have resulted in monkeys being less responsive to stressful environments later in life in our study [42]. In addition, HCCs in pair-housing were likely lower as we mostly selected maternally related pairs instead of forming new pairs with unrelated animals. HCCs in this study were thus relatively low compared to the other studies, even in pair-housing. However, due to the fact that HCCs in pair-housing were in general higher, it may not be possible to measure alterations in the same way between group-housed vs. pair-housed animals and the pair-housed control animals.

Still, the changes in HCC were highly variable, indicating that some individuals experienced less long-term stress and were indeed more resilient than others. We expected higher responsiveness to stress in monkeys with higher age, low dominance rank, and lower baseline HCCs. Yet, delta HCC was independent of these factors in our models. This is in contrast with reports that older animals and animals with lower baseline stress levels are generally more responsive to stress [22,26]. In addition, low-ranking long-tailed macaques are more vulnerable to a viral infection than high-ranking monkeys [27]. Since stress is known to suppress the immune function, this suggests that low-ranking animals are less resilient to stress compared to high-ranking individuals, but this was not found in our study. Based on changes in HCCs, we thus did not find individual characteristics associated with long-term stress when individuals enter an experiment.

Although social subordinates often have higher cortisol levels compared to dominants in NHPs [43,44,45,46], we found that HCCs were independent of dominance rank in both housing conditions. Control and predictability are commonly mentioned as important factors herein [22,47]. A meta-analysis by Abbott et al. (2003) showed that higher cortisol levels by subordinates are predicted by two factors: high stressor rates and low opportunities for social support [47]. The groups included in the present study contain several matrilines, in which females from the same matriline provide social support to each other. Besides, the enclosures in group- and pair-housing contain visual barriers and hiding places, which enables low-ranking females to avoid aggression by dominants, thereby having more control over their social interactions [22]. The lack of a correlation with dominance rank and the relatively low HCCs compared to other studies imply that both our housing conditions (group- and pair-housing) provide sufficient opportunities, at least for females, to cope with potential stressors.

Although HCCs were expected to correlate with body fat levels, this was not found. This contradicts with previous studies suggesting that long-term stress increases fat deposition in the abdominal area. These studies induced social stress by altering group composition, while in our study individuals were mostly paired with familiar individuals in a new environment. Only one formerly group-housed female was paired to an unfamiliar female and she had an extremely high pair-housed HCC. This suggests that a different social environment (i.e., cage mates) may be more stressful than a change in physical environment (i.e., enclosure). This may explain the lack of correlation between HCCs and body fat levels. In addition, the increase in HCC may have been too little or the time span too small to affect body fat levels in our study. Nevertheless, body fat levels did not reflect long-term stress in our study animals. Our results imply that changes in body fat levels may not always be suitable as biomarker for long-term stress. Nevertheless, they are still useful as indicators of health and welfare [7].

To determine body fat levels, both anthropometric measurements and CT analyses were used, a combination of external and internal measurements. Anthropometric measurements are a widely used and accepted method to estimate body fat levels in NHPs [48,49,50,51,52,53]. The use of CT to determine the percentage of body fat present around the abdomen is less common, though has been used before in both humans and NHPs [31,54,55,56,57,58]. The advantage of CT is that it provides information on the regional distribution of body fat to complement and validate the anthropometric measurements [59]. Although the two approaches yielded highly correlated measures, TBF and WHI produced slightly different results. TBF increased in 15 individuals, while WHI increased in only 13 individuals. However, WHI is based on body weight and height, which compromises more than body fat alone. Since smaller enclosures in pair-housing provide less space for physical activity, individuals may have lost some body weight due to loss of muscle tissue after the relocation. In addition, control over access to food may be different in pair-housing compared to group-housing. An increase in body fat counteracted by a decrease in muscle tissue may therefore result in a smaller increase, or even a decrease, in WHI compared to the TBF measured by CT.

Overall, body fat levels did not differ between housing conditions. This contrasts with the literature, where individually housed long-tailed macaques had higher body fat levels compared to group-housed monkeys [60]. Yet, there was considerable individual variation in body fat change between females. The change of housing conditions affected body fat alterations in a specific way depending on group-housed body fat level and group-housed dominance rank: lean and low-ranking individuals mainly gained body fat, while heavy and high-ranking individuals generally lost body fat. Monkeys with low group-housed body fat levels and dominance rank may have relatively low food intake in group-housing, while food is more equally divided in pair-housing. As a result, lean and low-ranking individuals may easily gain body fat, while heavy and high-ranking individuals may lose body fat in pair-housing.

Although high dominance rank has been associated with higher body fat and weight in other NHP studies [61,62], rank had no effect on body fat levels in this study. Our selection criteria may have introduced some bias here, as obese individuals were excluded from the study. Since group-housed dominance rank did affect the changes in body fat levels, group-housed dominance rank may be considered in animal selection when it is important that individuals do not gain/lose body fat. Low-ranking individuals should be preferred when animals should not lose body fat, while high-ranking individuals should be selected when the research requires animals not to gain much body fat. Based on the results of this study, this recommendation can be applied to females; however, it may be different for males.

## 5. Conclusions

In conclusion, HCCs significantly increased when female rhesus macaques were moved from group-to pair-housing, while average body fat levels did not differ. Changes in HCCs were independent of age, dominance rank, and group-housed HCC. There was large individual variation in body fat alterations, which was related to the group-housed body fat levels and dominance rank. However, there was no significant correlation between HCCs and body fat levels. This study did therefore not find individual characteristics related to long-term stress in pair-housing. However, the individual variation confirms that some individuals are more resilient than others and provides possibilities for future refinement studies.

## Data Availability

Data are available on request.

## Appendix A

**Table A1:** Spearman correlations between anthropometric measures and body fat percentages, measured by CT. n = 64, *p* < 0.0005. ^#^ Pearson correlation.

	WHI	AC	AST	ABF	SBF	TBF
BW	r = 0.857 ^#^	r = 0.945 ^#^	r = 0.847 ^#^	r = 0.859	r = 0.838	r = 0.894
WHI	X	r = 0.911 ^#^	r = 0.861 ^#^	r = 0.784	r = 0.855	r = 0.855
AC	X	X	r = 0.905 ^#^	r = 0.893	r = 0.847	r = 0.930
ASF	X	X	X	r = 0.846	r = 0.878	r = 0.892
ABF	X	X	X	X	r = 0.795	r = 0.977
SBF	X	X	X	X	X	r = 0.822

Abbreviations: BW = body weight (kg), WHI = weight-for-height index (kg/m^3^), AC = abdominal circumference (cm), AST = abdominal skinfold thickness (mm), ABF = abdominal body fat (%), SBF = subcutaneous body fat (%), TBF = total body fat (%).

**Table A2:** Outcome of the regression models for body fat change, as measured by all anthropometric and CT measures. Factors included in the models are age, group-housed dominance rank, pair-housed dominance rank, and the baseline value. n = 27. * 0.05 < *p* < 0.10, ** *p* < 0.05.

	Age	Group-Housed Dominance Rank	Pair-Housed Dominance Rank	Baseline Value
BW	F = 0.965, *p* = 0.337	F = 1.636, *p* = 0.214	F = 0.219, *p* = 0.644	F = 10.261, *p* = 0.004 **
WHI	F = 0.367, *p* = 0.551	F = 3.409, *p* = 0.078 *	F = 0.440, *p* = 0.514	F = 4.487, *p* = 0.046 **
AC	F = 0.535, *p* = 0.472	F = 4.048, *p* = 0.057 *	F = 0.106, *p* = 0.748	F = 8.764, *p* = 0.007 **
ASF	F = 1.296, *p* = 0.267	F = 3.526, *p* = 0.074 *	F = 0.044, *p* = 0.836	F = 9.229, *p* = 0.006 **
ABF	F = 1.697, *p* = 0.206	F = 4.594, *p* = 0.043 **	F = 0.005, *p* = 0.946	F = 10.936, *p* = 0.003 **
SBF	F = 0.019, *p* = 0.892	F = 2.433, *p* = 0.133	F = 0.144, *p* = 0.708	F = 4.033, *p* = 0.057 *
TBF	F = 0.955, *p* = 0.339	F = 4.282, *p* = 0.050 *	F = 0.010, *p* = 0.923	F = 9.373, *p* = 0.006 **

Abbreviations: BW = body weight (kg), WHI = weight-for-height index (kg/m^3^), AC = abdominal circumference (cm), AST = abdominal skinfold thickness (mm), ABF = abdominal body fat (%), SBF = subcutaneous body fat (%), TBF = total body fat (%).

**Table A3:** Checking the effect of potential confounding factors on the study’s major outcome measures with one-way ANOVA (F-value) and independent samples t-tests (t-value).

	Delta WHI (N = 27)	Delta TBF (N = 27)	Delta HCC (N = 26)
Number of procedures (0/5/8)	F = 0.397, *p* = 0.677	F = 0.862, *p* = 0.435	F = 0.365, *p* = 0.698
Number of injuries (0/1/2/3)	F = 0.132, *p* = 0.940	F = 0.288, *p* = 0.833	F = 1.801, *p* = 0.176
Exposure to humans (high vs. low)	t = 0.570, *p* = 0.575	t = 0.365, *p* = 0.719	t = −0.122, *p* = 0.912
Cage size (single vs. double)	t = 0.547, *p* = 0.590	t = 0.484, *p* = 0.633	t = 1.867, *p* = 0.074
Related pair (yes/no)	t = −0.181, *p* = 0.858	t = −0.369, *p* = 0.715	t = −0.950, *p* = 0.351
Trainability (high vs. low)	t = −0.801, *p* = 0.431	t = −1.988, *p* = 0.058	t = 0.573, *p* = 0.572

**Table A4:** Descriptive statistics on body fat measures in group-housing, pair-housing and body fat change. N = 27. Mean ± SE (minimum–maximum) are reported.

Measure	Group-Housing	Pair-Housing	Change
BW	7.9 ± 0.2 (5.9–10.1)	7.7 ± 0.2 (5.6–9.6)	−0.2 ± 0.2 (−2.5–1.7)
WHI	58.3 ± 1.4 (43.9–74.7)	56.3 ± 1.6 (41.1–71.7)	−2.0 ± 1.4 (−18.1–10.1)
AC	35.3 ± 1.0 (26.2–43.7)	35.0 ± 1.0 (26.9–44.4)	−0.3 ± 1.0 (−11.2–8.9)
ASF	9.9 ± 0.8 (3.0–17.9)	9.7 ± 1.0 (1.9–20.3)	−0.2 ± 1.0 (−12.7–9.5)
ABF	11.9 ± 1.9 (0–32.2)	14.8 ± 2.2 (0–40.5)	3.0 ± 2.4 (−26.6–35.1)
SBF	9.3 ± 1.1 (0–17.1)	10.0 ± 1.2 (0–20.7)	0.7 ± 1.2 (−13.9–11.5)
TBF	21.1 ± 2.8 (0–45.3)	24.8 ± 3.1 (0–48.3)	3.7 ± 3.4 (−38.5–39.0)

Abbreviations: BW = body weight (kg), WHI = weight−for−height index (kg/m^3^), AC = abdominal circumference (cm), AST = abdominal skinfold thickness (mm), ABF = abdominal body fat (%), SBF = subcutaneous body fat (%), TBF = total body fat (%).
